# Recovery from acute hypoxia: A systematic review of cognitive and physiological responses during the ‘hypoxia hangover’

**DOI:** 10.1371/journal.pone.0289716

**Published:** 2023-08-16

**Authors:** David M. Shaw, Peter M. Bloomfield, Anthony Benfell, Isadore Hughes, Nicholas Gant

**Affiliations:** 1 Aviation Medicine Unit, Royal New Zealand Air Force Base Auckland, Whenuapai, Auckland, New Zealand; 2 Department of Exercise Sciences, University of Auckland, Auckland, New Zealand; 3 Department of Medicine, University of Auckland, Auckland, New Zealand; Rutgers Robert Wood Johnson Medical School, UNITED STATES

## Abstract

Recovery of cognitive and physiological responses following a hypoxic exposure may not be considered in various operational and research settings. Understanding recovery profiles and influential factors can guide post-hypoxia restrictions to reduce the risk of further cognitive and physiological deterioration, and the potential for incidents and accidents. We systematically evaluated the available evidence on recovery of cognitive and basic physiological responses following an acute hypoxic exposure to improve understanding of the performance and safety implications, and to inform post-hypoxia restrictions. This systematic review summarises 30 studies that document the recovery of either a cognitive or physiological index from an acute hypoxic exposure. Titles and abstracts from PubMed (MEDLINE) and Scopus were searched from inception to July 2022, of which 22 full text articles were considered eligible. An additional 8 articles from other sources were identified and also considered eligible. The overall quality of evidence was moderate (average Rosendal score, 58%) and there was a large range of hypoxic exposures. Heart rate, peripheral blood haemoglobin-oxygen saturation and heart rate variability typically normalised within seconds-to-minutes following return to normoxia or hyperoxia. Whereas, cognitive performance, blood pressure, cerebral tissue oxygenation, ventilation and electroencephalogram indices could persist for minutes-to-hours following a hypoxic exposure, and one study suggested regional cerebral tissue oxygenation requires up to 24 hours to recover. Full recovery of most cognitive and physiological indices, however, appear much sooner and typically within ~2–4 hours. Based on these findings, there is evidence to support a ‘hypoxia hangover’ and a need to implement restrictions following acute hypoxic exposures. The severity and duration of these restrictions is unclear but should consider the population, subsequent requirement for safety-critical tasks and hypoxic exposure.

## Introduction

Hypoxia is a state of insufficient oxygen which can compromise normal physiological and cognitive functions, and manifests when breathing air with a lower partial pressure of oxygen (PO_2_) compared to sea-level (i.e. <159 mmHg) [1, 2]. The initial compensatory responses to the resulting hypoxaemia (i.e. low arterial partial pressure of oxygen) include cardiopulmonary, respiratory and metabolic, which aim to maintain oxygen supply to vital tissues, but tissues eventually desaturate when PO_2_ is sufficiently low. The brain’s high rates of oxidative metabolism (20–25% resting metabolic rate) make it vulnerable to oxygen depletion [3] and energetically-demanding cognitive functions are easily impaired during hypoxic exposures [1, 4]. Temporal recovery from hypoxia is assumed to be rapid since peripheral blood haemoglobin reoxygenates within seconds-to-minutes; however, cognitive and physiological perturbations can persist beyond the recovery of blood and tissue oxygenation [5]; a state that has been colloquially termed the ‘hypoxia hangover’ [6].

The recovery profiles of cognitive and physiological responses from an acute hypoxic exposure have performance and safety implications for various operational and research populations. For example, military aviators undertake hypoxia recognition training at least once every five years and are prohibited from flying duties for the following 12–24 hours. Recent studies by the Naval Medical Research Unit (Dayton, Ohio, USA), however, have demonstrated cognitive and physiological indices fully recover almost immediately [7] or within 2–4 hours [5] following a hypoxic exposure, which suggests the grounding period for military aviators could be reduced. If post-hypoxia restrictions are implemented, the influence of the hypoxic dose (barometric pressure, fraction of inspired oxygen [F_I_O_2_] and duration of exposure), recovery procedures (e.g. normoxic or hyperoxic breathing) and safety-critical nature of subsequent tasks should also be considered. There is a need to evaluate if post-hypoxia restrictions are required for different populations and to establish clear evidence-based recommendations that inform operational, training and research scenarios.

Therefore, we conducted a systematic review examining the recovery profiles of cognitive and physiological responses following an acute hypoxic exposure in healthy individuals. The aim was to determine whether post-hypoxia restrictions should be implemented, evaluate if the hypoxic dose or recovery procedures influence cognitive and basic physiological indices, and identify gaps in knowledge for future investigations. Outcomes will be crucial for populations that experience hypoxia during training or operational duty (e.g. military aviators), and to manage participants of research studies evaluating the effects of hypoxic interventions.

## Methodology

We followed the Preferred Reporting Items for Systematic Reviews and Meta-Analysis (PRISMA) 2020 guidelines [8]. This review was not preregistered as it stemmed from a project to inform recommendations following normobaric and hypobaric hypoxia recognition training of military aviators within the Royal New Zealand Air Force.

### Search

**Fig 1:**
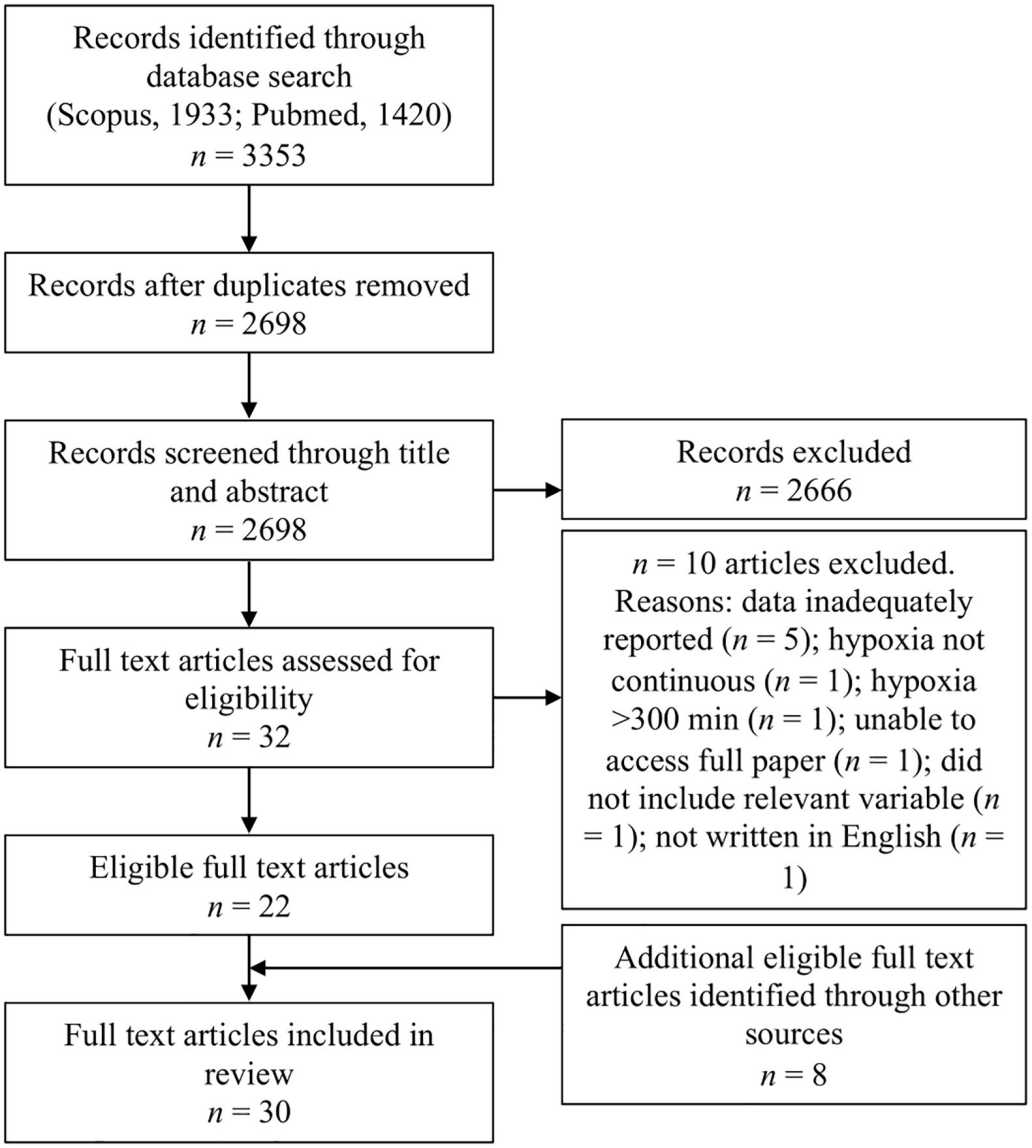
PRISMA flow chart.

We searched titles and abstracts from PubMed (MEDLINE) and Scopus from inception to July 2022 for potential research studies using the search terms and Boolean operators: (hypoxia or hypoxic) AND (hangover OR recovery) AND (cognition OR cognitive OR "cognitive performance" OR “flight performance” OR mood OR sleepiness OR symptoms OR effort OR physiology OR physiological OR oxygenation OR "oxygen saturation" OR "heart rate" OR "heart rate variability" OR “blood pressure” OR ventilation OR “respiratory rate” OR respiration OR “blood gases”) NOT (animal OR murine OR rodent OR mice OR porcine OR dog OR fish OR cell). Furthermore, additional articles known to the first author (DMS) that were not identified in the literature search were added and titles (only) of the following were screened by DMS: 1) reference lists of eligible articles; 2) forward citation tracking using Google Scholar of eligible articles; and 3) searching key journals and Google Scholar using a combination of the terms: hypoxia, hypoxic, recovery and hangover. All potential articles identified through other sources were screened by DMS immediately following retrieval. Full details of the screening process are displayed in Fig 1.

### Inclusion and exclusion criteria

We included studies fulfilling the following criteria: 1) between- or within-subject experimental trials; 2) human participants of any age with no known medical conditions; 3) an objective measure of cognition or physiology during recovery from an acute hypoxic exposure; and 4) peer reviewed full text original research studies published in English. *Acute hypoxia* was defined as <300 min of breathing hypoxic air that lowered peripheral blood haemoglobin-oxygen saturation (SpO_2_) to <90% (i.e. normobaric or hypobaric hypoxia) and *recovery* was defined as the period of breathing air that increased and normalised peripheral blood SpO_2_ to >95% following an acute hypoxic exposure. We excluded studies if: 1) the control or baseline group (or condition) were not administered air able to maintain SpO_2_ >95%; 2) other interventions known to effect cognition were included in conjunction with oxygen manipulation, except for carbon dioxide; 3) single or accumulated repeated hypoxia exposures were administered beyond 300 min; 4) hypoxia was repeatedly interrupted by normoxic breathing; and 5) cognitive and/or physiological data were not adequately reported. As we were specifically interested in physiological responses that can be readily measured in military aviation training and operational settings, we restricted our physiological indices of interest to ventilatory, cardiovascular, regional cerebral tissue haemoglobin-oxygen saturation (rSO_2_), SpO_2_, and cardiac autonomic responses. Measures of less interest and low feasibility in the training and operational setting (e.g. intra-ocular pressure, limb blood flow, and vascular resistance) were omitted. Review articles, unpublished abstracts, theses, and dissertations were also excluded.

### Screening

Literature search results were entered into Mendeley, which automatically removed duplicates, then exported to Rayyan (i.e. online systematic review software). Two authors (DMS and AB) independently screened titles and abstracts for suitability. The full text for studies of interest were retrieved and independently evaluated for suitability by the same two authors. Disagreements between authors’ decisions were resolved via discussion and consensus.

### Risk of bias/quality assessment

Two authors (DMS and PMB) independently performed the assessment of risk of bias in the included studies using the Rosendal Scale [9]. This scale combines the PEDro scale [10], Jadad scoring system [11] and Delphi list [12]. This Rosendal scale was selected as the PEDro scale, Jadad scoring system and Delphi list have been extensively evaluated and validated. A Rosendal score of 60% is considered as excellent methodological quality [9]. Two checklist items were removed, including to whether the researchers were blinded as it was deemed inappropriate (i.e. due to safety reasons) and reporting of methods used to report adverse effects as the responses to acute hypoxia are inherently deemed adverse. An additional checklist item assessing whether treatment order was counterbalanced was included. No studies were excluded based on quality assessment results. Disagreements were resolved by discussion.

## Results

### Overview of included studies and study quality

The literature search initially identified 2698 studies for screening. The full text of 32 studies were assessed and 22 were considered eligible. An additional 8 studies from other sources were identified and considered eligible, giving a total of 30 full text articles included in this review. Table 1 provides an overview of hypoxia and recovery interventions for the included studies. 20 studies utilised poikilocapnic hypoxia [5, 6, 13–30], 9 studies utilised isocapnic hypoxia [31–39] and 1 study utilised hypercapnic hypoxia [40]. The duration of hypoxia was typically <30 min and depended on the severity of hypoxic breathing; for instance, studies assessing ~25,000 ft (7,600 m) equivalent (i.e. ~7–8% oxygen) were ~5 min or less, whereas, studies assessing ~18,000 ft (5,486 m) equivalent (i.e. ~10–11% oxygen) were ~25–30 min. The severity of hypoxic breathing was generally >18,000 ft equivalent or <10–11% oxygen, and some studies titrated the F_I_O_2_ to maintain a specific end-tidal partial pressure of oxygen (P_ET_O_2_) or SpO_2_. Some studies assessed different hypoxic durations and severities. For recovery, 19 studies only administered normoxia (i.e. 21% oxygen) [14, 15, 20–27, 30, 32–34, 36–40], 2 studies only administered hyperoxia [18, 29], 5 studies administered both normoxia and hyperoxia [6, 13, 17, 28, 35], and 4 studies compared differences between normoxic and hyperoxic interventions [5, 16, 19, 31]. We included measures of cognitive performance, SpO_2_, heart rate (HR), heart rate variability (HRV; specifically, root mean square of successive differences between normal heartbeats [rMSSD], standard deviation of NN intervals [SDNN] and low-frequency [LF]/high-frequency [HF] ratio of spectral domains), blood pressure (BP; specifically mean arterial pressure [MAP], diastolic blood pressure [DBP] and systolic blood pressure [SBP]), rSO_2_, ventilation and electroencephalogram (EEG) measurements. Methodological quality assessment yielded an average Rosendal score of 58 ± 15% (range, 30–87%). Full results of the quality assessment can be found in S1 Table.

**Table 1:** Characteristics of included studies reporting recovery of cognitive and/or physiological responses following a hypoxic exposure.

Reference	Population	Hypoxic and Recovery Interventions
Bascom et al. (1992)	*n* = 6 (4 men, 2 women), mean age = 24.5, healthy adults	*Method*: Mouth-piece *Baseline*: 10 min of normobaric O_2_ to P_ET_O_2_ of 100 mmHg, with P_ET_CO_2_ held at 1–2 mmHg above resting *Hypoxia*: 20 min of reduced normobaric O_2_ to P_ET_O_2_ of 45, 50, 55, 65 or 75 mmHg, with P_ET_CO_2_ held at 1–2 mmHg above resting *Recovery*: 5 min of normobaric O_2_ to P_ET_O_2_ of 100 mmHg, with P_ET_CO_2_ held at 1–2 mmHg above resting *Note*: The second hypoxia period was omitted.
Beer et al. (2017)	*n* = 11 (9 men, 3 women; *n* = 1 dropout but sex not specified), healthy military personnel	*Method*: Hypobaric chamber and mask *Baseline*: 15 min of normobaric 21% O_2_, then ascent to 10,000 ft at 5,000 ft/min, 9 min of 10,000 ft, then 30 min of 100% O_2_ at 10,000 ft *Hypoxia*: Ascent to 18,000 ft or 25,000 ft at 5000 ft/min, then maintained for 20 min or (typically) <5 min, respectively *Recovery*: 100% O_2_ during descent to ground level at 5000 ft/min for SpO_2_ >90%, then 15 min of normobaric 21% O_2_ *Note*: Mask-on only during 100% O_2_
Blacker et al. (2021)	*n* = 26 (14 men, 12 women; *n* = 3 excluded due to noisy EEG data but sex not specified), mean age = 30.5, healthy Air Force personnel	*Method*: Reduced oxygen breathing environment and mask *Baseline*: 30 min normobaric 21% O_2_ *Hypoxia*: 10 min normobaric 9.7% O_2_ (~20,000 ft equivalent) *Recovery*: 30 min normobaric 21% or 100% oxygen, then 210 min (mask-off) normobaric 21% O_2_
Botek et al. (2015)	*n* = 29, men, mean age = 26, healthy sports science students	*Method*: Mask *Baseline*: 7 min (mask off) normobaric 21% O_2_ *Hypoxia*: 10 min normobaric 9.6% O_2_ (~20,341 ft equivalent) *Recovery*: 7 min (mask-off) normobaric 21% O_2_
Botek et al. (2018)	*n* = 58 (28 men, 30 women), mean age = 23.8, healthy students	*Method*: Mask *Baseline*: 6 min (mask off) normobaric 21% O_2_ *Hypoxia*: 10 min normobaric 9.6% (~20,341 ft equivalent) *Recovery*: 7 min normobaric 21% O_2_
Dahan et al. (1995)	*n* = 10 (6 men, 4 women), age range = 23 to 32, healthy adults	*Method*: Mask *Baseline*: 15–20 min normobaric O_2_ to P_ET_O_2_ 109 mmHg (i.e. normoxic) or normobaric O_2_ to P_ET_O_2_ 525 mmHg (i.e. hyperoxic) *Hypoxia*: 30 sec or 1, 3, and 5 min reduced normobaric O_2_ to P_ET_O_2_ 49 mmHg *Recovery*: 3–5 min normobaric 21% O_2_ (3 and 5 min hypoxic exposures were administered an additional 10 min normobaric 70% O_2_) or 3–5 min hyperoxia (P_ET_O_2_ 525 mmHg)
Dart et al. (2017)	*n* = 10, men, mean age = 31.4, healthy military personnel	*Method*: Hypobaric chamber and mask *Baseline*: 15 min normobaric 100% O_2_ prior to the 10,000 ft and 15,000 ft exposures and 30 min normobaric 100% O_2_ prior to the 20,000 ft exposure *Hypoxia*: ~60 min, ~45 min, and ~20 min at 10,000 ft, 15,000 ft, and 20,000 ft, respectively *Recover*y: 100% O_2_ during descent to ground level, then 1 hour (mask-off) 21% normobaric O_2_ *Note*: Mask-on only during 100% O_2_
Easton et al. (1988)	*n* = 11 (5 men, 6 women), mean age = 25, healthy adults	*Method*: Mouth-piece *Baseline*: 6–8 min normobaric 21% O_2_ *Hypoxia*: 25 min normobaric 8–10% O_2_ to SpO_2_ of ~80% with P_ET_CO_2_ held constant to baseline *Recovery*: Either; 1) 7 min normobaric 21% O_2_; 2) 15 min normobaric 21% O_2_; 3) 60 min normobaric 21% O_2_; 4) 7 min normobaric 100% O_2_; or 5) 15 min normobaric 30% O_2_, *Note*: The second hypoxic period was omitted
Georgopoulos et al. (1990)	*n* = 8 (5 men, 3 women), mean age = 28, healthy adults	*Method*: Mouth-piece *Baseline*: 8–10 min normobaric 21% O_2_, then 5 min 21% O_2_ with added CO_2_ (i.e. F_I_CO_2_ of 4.5–5%; P_ET_CO_2_ 5–14 mmHg), then 5 min 21% O_2_ *Hypoxia*: 25 min normobaric 8–9% O_2_ to SpO_2_ of ~80% (or 21% O_2_) with P_ET_CO_2_ held constant to baseline *Recovery*: 5 min normobaric 21% O_2_, then 5 min 21% O_2_ with added CO_2_ (i.e. CO_2_ titrated to match P_ET_CO_2_ of initial hypercapnic exposure), then 5 min 21% O_2_
Harshman et al. (2015)	*n* = 8, men, mean age = 29.9, healthy military personnel	*Method*: Mask *Baselin*e: 5 min normobaric 21% O_2_ *Hypoxia*: 5 min normobaric 8% O_2_ *Recovery*: 5 min normobaric 100% O_2_
Janaky et al. (2007)	*n* = 14, men, mean age = 39.7, health adults, either candidates for pilot school or lab personnel	*Method*: Hypobaric chamber *Baseline*: 20 min normobaric 21% O_2_ *Hypoxia*: 15 min at 5500 m (18,044 ft) *Recovery*: 20 min normobaric 21% O_2_
Malle et al. (2016)	*n* = 42, men, mean age = 29.4, healthy adults	*Method*: Mask *Baseline*: 30 sec normobaric 21% O_2_ or 100% O_2_ (normoxia included 10 min prior 21% O_2_ and hyperoxia included 3 min prior 100% O_2_) *Hypoxia*: ~3 min normobaric 6% O_2_ (or 21% O_2_) *Recovery*: 10 min normobaric 21% O_2_ or 3 min 100% O_2_ then 21% O_2_ *Note*: n = 22 in Hypoxia-Air group and *n* = 20 in Hypoxia-O_2_ group; comparisons between group and to baseline.
Morgan et al. (1995)	*n* = 10, men, mean age = ~37, healthy adults	*Method*: Mask *Baseline*: Normobaric 21% O_2_ *Hypoxia*: 20 min reduced normobaric O_2_ to SpO_2_ 80% and increased CO_2_ to P_ET_CO_2_ to +5 mmHg of baseline (control group of *n* = 5 breathed 21% O_2_ for 40 min) *Recovery*: 20 min normobaric 21% O_2_
Najmanová et al. (2019)	*n* = 38 (15 men, 23 women), mean age = ~25, healthy adults	*Method*: Mask *Baseline*: 7 min normobaric 21% O_2_ *Hypoxia*: 10 min normobaric 9.6% O_2_ (~20,341 ft equivalent) *Recovery*: 7 min normobaric 21% O_2_
Phillips et al. (2009)	*n* = 36, healthy military aviation personnel	*Method*: Mask *Baseline*: 10 min 21% O_2_ *Hypoxia* (profile 1, *n* = 9): 3 rapid (2–3 s) transitions to normobaric 9.1, 11.4 and 14.1% O_2_ with rapid return to 21% O_2_ between each (i.e. 5 min at altitude and 5 min at 21% O_2_), then gradual transition (4 min) to normobaric 9.1% O_2_ and return to 21% O_2_ *Hypoxia* (profile 2, *n* = 7): Gradual transition (4 min) to normobaric 9.1% O_2_ and return to 21% O_2_, then 3 rapid (2–3 s) transitions to normobaric 9.1, 14.1, and 11.4% O_2_ with return to 21% O_2_ between each (i.e. 5 min at altitude and 5 min at 21% O_2_) *Note*: *n* = 20 were in the control (normoxic) group. *Recovery*: Gradual (4 min) transition to normobaric 21% O_2_, then maintained for 10 min
Phillips et al. (2015)	*n* = 19, healthy active duty military personnel	*Method*: Mask *Baseline*: 21% normobaric O_2_ on prior visit *Hypoxia*: 30 min (or until SpO_2_ dropped below 50%) normobaric 9.96% O_2_ (~18,000 ft equivalent) *Recovery*: 24 hr normobaric 21% O_2_
Querido et al. (2010)	*n* = 7 (5 men, 2 women), mean age = 30, healthy	*Method*: Mask *Baseline*: ≥15 min normobaric 21% O_2_ *Hypoxia*: 20 min reduced O_2_ to SpO_2_ 80% and increased CO_2_ to P_ET_CO_2_ to baseline levels *Recovery*: ~4.5 min normobaric 21%, then 1 min normobaric 100% O_2_ (termed “intervention” and repeated 3 times)
Querido et al. (2011)	*n* = 14 (11 men, 3 women), mean age = 26, healthy adults	*Method*: Mask *Baseline*: 15 min normobaric 21% O_2_ *Hypoxia*: 20 min of reduced normobaric O_2_ to SpO_2_ of 80% with P_ET_CO_2_ maintained at baseline levels *Recovery*: ~5 min normobaric 21% O_2_ (n = 15) and 20 min normobaric 21% O_2_ (*n* = 5)
Robinson et al. (2018)	*n* = 21 (20 men, 1 women), age range = 22–37, healthy Air Force personnel (non-pilots)	*Method*: Mask *Baselin*e: 5 min normobaric 21% O_2_ *Hypoxia*: 5 min normobaric 6.5% O_2_ (~25,000 ft equivalent) *Recovery*: 5 min normobaric 21% O_2_ followed by 30 min normobaric 21% or 13.1% O_2_ (~10,000 ft equivalent) *Note*: Comparisons with baseline were not possible due to methodological issues
Roche et al. (2002)	*n =* 11 (6 men, 5 women), mean age = 29, healthy adults	*Method*: Mask *Baseline*: 20 min normobaric 21% O_2_ *Hypoxia*: 15 min normobaric 11% O_2_ (~15,748 ft equivalent) *Recovery*: 20 min normobaric 21% O_2_
Sausen et al. (2003)	*n* = 4 (3 men, 1 women), mean age = 24.7, healthy US student Naval flight surgeons	*Method*: Mask *Baseline*: 15 min normobaric 21% O_2_ *Hypoxia*: 20 min transition to normobaric 10% O_2_ (~18,000 ft equivalent), then 30 min 10% O_2_, then 5 min transition to normobaric 21% O_2_ *Recovery*: 15 min normobaric 21% O_2_
Steinback et al. (2012)	*n* = 7 (3 men, 4 women), mean age = 27, healthy adults	*Method*: Mask *Baseline*: 10 min normobaric 21% O_2_ *Hypoxia*: Step decrease in normobaric O_2_ to P_ET_O_2_ of 45 mmHg (~80% SpO_2_) and maintenance of baseline P_ET_CO_2_, then maintained for 5 min *Recovery*: 10 min normobaric 21% O_2_
Stepanek et al. (2013)	*n* = 25 (14 men, 11 women), mean age = 32.4, healthy adults	*Method*: Mask *Baseline*: Normobaric 21% O_2_ *Hypoxia*: ~4–5 min normobaric 8% O_2_ (~23,300 ft equivalent) *Recovery*: ~4–5 min normobaric 21% O_2_
Stepanek et al. (2014)	*n* = 25 (14 men, 11 women), mean age = 32.4, healthy adults	*Method*: Mask 2 profiles separated by a 6 min washout period *Baseline*: Normobaric 21% O_2_ *Hypoxia*: 3 min of either normobaric 8% O_2_ or normobaric 7% O_2_ + 5% CO_2_ *Recovery*: 3 min normobaric 21% O_2_
Tamisier et al. (2005)	*n* = 10 (6 men, 4 women), mean age = 29.8, healthy adults	*Method*: Mask Baseline: Normobaric 21% O_2_ Hypoxia: 2 hr normobaric ~9% O_2_ to ~85% SpO_2_ Recovery: Normobaric 21% O_2_
Uchida et al. (2020)	*n* = 9 (2 men, 7 women), mean age = 28, healthy adults	*Method*: Mask *Baseline*: 7 min normobaric 21% O_2_ *Hypoxia*: 7–10 min normobaric 11.8% O_2_ (~15,00 ft equivalent) *Recovery*: 13–16 min normobaric 21% O_2_
Varis et al. (2019)	*n* = 16, men, healthy qualified Hawk pilots	*Method*: Mask 3 profiles separated by 10 min washout periods within ~40 min *Baseline*: N/A *Hypoxia*: Pressurised air, then ~123 sec, ~93 sec, and ~115 sec of normobaric 8% (~20,341 ft equivalent), 7% (~22,966 ft equivalent) or 6% O_2_ (~25,919 ft equivalent), respectively *Recovery*: 100% normobaric O_2_ until emergency procedure completion; return to base flight 10 min after emergency procedures completion *Note*: Performance was compared to control flight with normobaric 21% O_2_ (not hypoxia) following the 6% O_2_ condition only; 60 minutes rest between trials
Varis et al. (2022)	*n* = 15, men, mean age = 24.6, healthy Hawk fighter pilots	*Method*: Mask *Baseline*: N/A *Hypoxia*: ~75 sec normobaric 6% O_2_ or ~103 sec normobaric 8% O_2_ *Recovery*: ~58 sec normobaric 100% O_2_ following 6% O_2_ and ~42 sec normobaric 100% O_2_ following 8% O_2_ (to complete emergency procedures); return to base flight 10 min after emergency procedures completion *Note*: Performance was compared to control flight with normobaric 21% O_2_ (not hypoxia)
Vigo et al. (2010)	*n* = 12, men, mean age = 28, healthy military pilots	*Method*: Hypobaric chamber and mask *Baseline*: ~89 sec 100% O_2_ during ascent to 27,000 ft *Hypoxia*: ~113 sec at 27,000 ft *Recovery*: ~137 sec 100% O_2_ at 27,000 ft
Xie et al. (2001)	*SpO2*, *HR*, *BP and HRV*: *n* = 11 (7 men, 4 women), mean age = 26, healthy adults *Ventilation*: *n* = 9 (6 men, 3 women), mean age = 29, healthy adults	*Method*: Mask *Baseline*: 10 min normobaric 21% O_2_ *Hypoxia*: 20 min normobaric 10–12% O_2_ to lower SpO_2_ to ~80% and maintain baseline P_ET_CO_2_ *Recovery*: 20 min normobaric 21% O_2_

The estimated altitude equivalent is reported for studies using normobaric hypoxia that reported targeting a specific altitude. Altitude equivalents are reported in ft in line with aviation standards; for reference: 5,000 ft = 1,524 m, 10,000 ft = 3,048 m, 18,000 ft = 5,486 m, and 25,000 ft = 7,620 m. Abbreviations: O_2_ = oxygen; SpO_2_ = peripheral blood haemoglobin-oxygen saturation; P_ET_O_2_ = end-tidal partial pressure of oxygen; CO_2_ = carbon dioxide; F_I_CO_2_ = fraction of inspired carbon dioxide; P_ET_CO_2_ = end-tidal partial pressure of carbon dioxide.

### Cognition

Eleven studies measured cognition during recovery from hypoxia, including measures from standardised tests and simulated flight performance (Table 2). One study measured composite scores of different cognitive domains within the synthetic work environment (SYNWIN) test battery and reported normalisation at ~20 min [13]. Three studies measured simple and choice reaction time; 1 study reported normalisation at 60 min [17] and 2 studies reported continued impairments, 1 at 10 min [21] and 1 at 24 hours [20]. Two studies measured attention; 1 study reported normalisation of reaction time and lapses (using the psychomotor vigilance task [PVT]) at 60 min [5] and 1 study reported normalisation of commission errors (using the Conners’ continuous performance test) at 13–16 min [27]. One study measured sequential number reading and reported normalisation at 5 min [25] and 1 study measured mathematical processing using the Paced Auditory Serial Addition Task (PASAT), a proxy of attention and working memory, and reported normalisation at 90 sec [19]. Two studies measured simulated flight performance and reported impairments for 10 min [6, 28]. Another study also measured simulated flight performance, in conjunction with a time-estimation task, but due to methodological issues, could not make comparisons to baseline; instead, comparisons between 21% and 13.1% oxygen recovery revealed no difference in flight performance errors, and time-estimation task lapses and variance at 35 min [22]. Not all hypoxic protocols impaired all measures of cognition performance [5, 20, 27] and 21% versus 100% oxygen did not elicit clear or persistent differences in cognitive performance [5, 19].

**Table 2:** Studies reporting recovery of cognitive responses following a hypoxic exposure.

Reference	Cognitive task	Effect of hypoxia	Recovery from hypoxia
Beer et al. (2017)	SYNWIN test battery (short-term memory, mathematical addition, visual monitoring, auditory monitoring)	Reduced composite test battery scores (18,000 ft scores <25,000 ft scores)	Composite test battery scores normalised at ~20 min
Blacker et al. (2021)	10 min psychomotor vigilance task (median reaction time and minor [500–1000 ms] and major [>1000 ms] lapses)	Slower median reaction time and (probably) increased minor lapses but no effect for major lapses	Median reaction time and minor lapses normalised at 60 min for 21% and 100% O_2_ (slower at 20 min)
Dart et al. (2017)	Simple and choice reaction time tasks	Slower total response time for simple and choice reaction time tasks at 15,000 and 20,000 ft but not 10,000 ft; negligible differences in accuracy for simple and choice reaction time tasks	Total response time for simple and choice reaction tasks normalised at 60 min
Malle et al. (2016)	PASAT (attentional processing and working memory)	Reduced correct responses, increased omissions, increased errors and increased miscalculations	*Correct responses, omissions, errors and miscalculations normalised at 80 sec for 21% and 100% O_2_; however, 100% O_2_ had initial transient lower correct responses and increased omissions compared to 21% O_2_
Phillips et al. (2009)	Flanker arrow task (two choice reaction time i.e. response conflict alone and stimulus + response conflict) *(during the 9*.*1% O*_*2*_ *trial)*	Slower 2-choice reaction time	Slower 2-choice reaction time at 10 min
Phillips et al. (2015)	Cognitive/Perceptual test battery (Number Stroop Task, reaction time, choice reaction time, NASA-TLX)	Slower simple and choice reaction time, increased task effort but no effect for Number Stroop task	Simple and choice reaction time normalised at 24 hours (60 and 120 min remained increased) and task effort normalised immediately
Robinson et al. (2018)	Simulator flight performance and time estimation task	Increased flight performance errors, time-estimation task lapses and variance	*No difference between 13.1% and 21% O_2_ for flight performance errors, and time-estimation task lapses and variance at 35 min
Stepanek et al. (2013)	King-Devick test (sequential rapid number reading)	Slower task completion and increased errors	Task time and errors normalised at 5 min
Uchida et al. (2020)	Conners’ continuous performance test–(reaction time and commission and omission errors)	Increased commission errors but no effect on reaction time or omission errors	Commission errors normalised at 13–16 min
Varis et al. (2019)	Simulator flight performance	Unclear, but likely impaired situational awareness and flight performance	Impaired situational awareness and flight performance at ~10 min; adverse subjective effects reported up to 12 hours after, such as fatigue, tiredness, dizziness and headaches
Varis et al. (2022)	Simulator flight performance	Unclear, but likely impaired flight performance	Impaired flight performance following 6% (but not 8%) O_2_ at ~10 min; adverse subjective effects reported immediately after, such as light headedness, visual impairments and dizziness

Effects are compared to a normoxic baseline (i.e. either immediately before the hypoxic intervention or during a separate trial), unless otherwise indicated.

*Comparisons were between conditions (not to baseline). Altitude equivalents are reported in ft in line with aviation standards; for reference: 5,000 ft = 1,524 m, 10,000 ft = 3,048 m, 18,000 ft = 5,486 m, and 25,000 ft = 7,620 m. Abbreviations: SYNWIN = Synthetic work environment; PASAT = Paced Auditory Serial Addition Task; O_2_ = oxygen; NASA-TLX = National Aeronautics and Space Administration-Task Load Index.

### Cardiovascular responses

Table 3 provides an overview of the recovery of SpO_2_, HR, HRV and BP during recovery from hypoxia. Eighteen studies measured SpO_2_; SpO_2_ normalised within 1–10 minutes in almost all studies [5, 14, 15, 18, 19, 21, 24–26, 29, 34–37] and three studies reported SpO_2_ normalised by 13–35 min [23, 27, 30]. One study could only report SpO_2_ recovery compared to a hypoxic condition [22]. Fifteen studies measured HR; HR recovered in 1–10 min in nine studies [5, 19, 24, 29, 33, 35, 36, 38, 39] and 11–20 min in three studies [26, 27]. HR was lower at 7 min in two studies [14, 15] and one study could only report HR recovery compared to a hypoxic condition [22]. Five studies measured HRV indices; HRV normalised at 3–7 min in three studies [14, 15, 19] and at 20 min in one study [23], whereas one study reported some perturbations of HRV persisted at ~137 sec [29]. Seven studies measured BP indices; BP normalised at 5–10 min in 3 studies (i.e. SBP [33, 38] and MAP and SBP [39]), and SBP and DBP normalised at 20 min in one study [30]. However, SBP remained increased after an unspecified time in one study [35], DBP and SBP remained increased at 5 min in one study [36], and MAP remained increased at 11 min in one study [26]. HR, HRV and BP indices were not altered by all hypoxic interventions, and 100% oxygen breathing during recovery normalised SpO_2_ [5, 19] and HR [5] only marginally faster (seconds) than 21% oxygen.

**Table 3:** Studies reporting recovery of cardiovascular responses following a hypoxic exposure.

Reference	Effect of hypoxia	Recovery from hypoxia
Blacker et al. (2021)	Reduced SpO_2_ and increased HR	SpO_2_ normalised at 2 min with 100% O_2_ and 5 min with 21% O_2_; HR normalised at 2 min with 21% and 100% O_2_
Botek et al. (2015)	Reduced SpO_2_, increased HR and increased (natural logarithm) LF/HF ratio	SpO_2_ and (natural logarithm) LF/HF HRV normalised and HR reduced at 7 min
Botek et al. (2018)	Reduced SpO_2_, increased HR, reduced log rMSSD and SDNN (both sexes), increased LF/HF (males only)	SpO_2_ normalised at 7 min in females, but remained reduced in males; HR reduced at 7 min in both sexes; log rMSSD (both sexes), SDNN (males only) and LF/HF (both sexes) normalised at 7 min
Harshman et al. (2015)	Reduced SpO_2_	SpO_2_ normalised at ~1 min
Janáky et al. (2007)	Reduced SpO_2_, increased SBP and DBP, but no effect for HR	SpO_2_,, SBP and DBP normalised at 20 min
Malle et al. (2016)	Reduced SpO_2_, increased HR and reduced HRV	*SpO_2_, HR and HRV normalised at ~3 min; albeit SpO_2_ increased more rapidly with 100% O_2_
Morgan et al. (1995)	Increased HR and SBP, but no effect for DBP	HR and SBP normalised at 5 min
Najmanová (2019)	Reduced SpO_2_ (greater reduction in women)	SpO_2_ normalised at 7 min
Phillips et al. (2009)	Reduced SpO_2_	SpO_2_ normalised at ~1 min
Querido et al. (2010)	Reduced SpO_2_ and increased HR and SBP, but no effect for DBP and MAP	SpO_2_ and HR normalised at ~5 min (i.e. with 21% O_2_), but SBP remained increased at ~15 min (i.e. following 3^rd^ intervention period) (no effect of hyperoxia vs normoxia)
Querido et al. (2011)	Reduced SpO_2_ and increased HR, MAP, SBP and DBP	SpO_2_ and HR normalised at 5 min, and MAP, SBP and DBP remained increased at 5 min
Roche et al. (2002)	Increased HR and LF/HF ratio, and reduced SpO_2_, but no effect for SBP, DBP and mean BP	HR, LF/HF ratio and SpO_2_ normalised at 20 min
Robinson et al. (2018)	Reduced SpO_2_ and increased HR	*SpO_2_ lower and HR higher in 13.1% compared with 21% following hypoxia at 35 min; no difference in SpO_2_ or HR during 13.1% O_2_ when preceded by hypoxia or normoxia
Sausen et al. (2003)	Reduced SpO_2_ and increased HR, but no effect for MAP, SBP and DBP	SpO_2_ normalised at 2 min and HR normalised at 6 min
Steinback et al. (2012)	Increased HR, MAP and SBP, but no effect for DBP	HR, MAP and SBP normalised at 10 min
Stepanek et al. (2013)	Reduced SpO_2_	SpO_2_ normalised at 2 min
Stepanek et al. (2014)	Reduced SpO_2_	SpO_2_ normalised at 3 min following both exposures
Tamisier et al. (2005)	Reduced SpO_2_ (BP and HR not measured)	SpO_2_ normalised at 1 min; BP increased at 11 min; HR normalised at 11 min
Uchida et al. (2020)	Reduced SpO_2_ and increased HR, but no effect for MAP	SpO_2_ and HR normalised at 13–16 min
Vigo et al. (2010)	Reduced SpO_2_, increased HR, reduced SDNN, but no effect for rMSSD and LF/HF	SpO_2_ and HR normalised at ~137 sec, but SDNN remained reduced
Xie et al. (2001)	Increased HR and SBP, but no effect for DBP	HR normalised at 5 min and SBP normalised at 10 min

Effects are compared to a normoxic baseline (i.e. either immediately before the hypoxic intervention or during a separate trial), unless otherwise indicated.

*Comparisons were between conditions (not to baseline). Abbreviations: SpO_2_ = peripheral blood haemoglobin oxygen saturation; HR = heart rate; HRV = heart rate variability; SDNN = standard deviation of RR intervals; rMSSD = root mean square of successive differences between normal heartbeats; LF = low-frequency power; HF = high-frequency power; MAP = mean arterial pressure; SBP = systolic blood pressure; DBP = diastolic blood pressure.

### Ventilatory responses

Ten studies measured ventilatory indices during recovery from hypoxia (Table 4). Minute ventilation (VE) normalised within seconds to 15 min in almost all studies [16, 27, 31–33, 35, 36, 38, 39]; however, one study reported VE was reduced after 5 min [40] and one study reported VE during hyperoxic recovery transiently undershot hyperoxic baseline following 3 and 5 min of hypoxia (but not shorter hypoxia exposures) [16].

**Table 4:** Studies reporting recovery of ventilatory responses following a hypoxic exposure.

Reference	Effect of hypoxia	Recovery from hypoxia
Bascom et al. (1992)	Increased VE	Slightly reduced VE at 5 min for all hypoxia levels
Dahan et al. (1995)	Increased VE	VE normalised at 69, 54, 12 and 12 sec when 30 sec and 1, 3 and 5 min of hypoxia, respectively, were followed by 21% O_2_; hyperoxic VE normalised (relative to hyperoxic baseline) at 9, 15, 12 and 9 sec following 30 sec and 1, 3 and 5 min of hypoxia, respectively; hyperoxic VE transiently undershot hyperoxic baseline following 3 and 5 min of hypoxia
Easton et al. (1988)	Increased VE	VE normalised at 60 min (almost normalised at 15 min) for 21% O_2_, 15 min for 30% O_2_ and 7 min for 100% O_2_
Georgopoulos et al. (1990)	Increased VE and tidal volume, but no effect for breathing frequency	VE and tidal volume normalised at 5 min (hypoxia did not affect the ventilatory response to hypercapnia)
Morgan et al. (1995)	Increased VE	VE normalised at 5 min
Querido et al. (2010)	Increased VE and tidal volume, but no effect for breathing frequency	VE and tidal volume normalised at ~5 min (i.e. with 21% O_2_)
Querido et al. (2011)	Increased VE and breathing frequency, but no effect for tidal volume	VE and breathing frequency normalised at 5 min
Steinback et al. (2012)	Increased VE and tidal volume, but no effect for breathing frequency	VE and tidal volume normalised at 10 min
Uchida et al. (2020)	Increased VE	VE normalised at 13–16 min
Xie et al. (2001)	Increased VE	VE normalised at 5 min

Effects are compared to a normoxic baseline (i.e. either immediately before the hypoxic intervention or during a separate trial), unless otherwise indicated. Abbreviations: VE = minute ventilation; O_2_ = oxygen.

### Neurophysiological responses

#### Cerebral tissue oxygenation

Four studies measured rSO_2_ during recovery from hypoxia (Table 5). rSO_2_ normalised within 10–13 min in two studies [27, 39], whereas one study reported rSO_2_ did not normalise after 10 min [20] and one study reported rSO_2_ normalised at 24 hours, but not at 2 hours [20].

**Table 5:** Studies reporting recovery of regional cerebral tissue oxygenation responses following a hypoxic exposure.

Reference	Effect of hypoxia	Recovery from hypoxia
Phillips et al. (2009)	Reduced rSO_2_	Reduced rSO_2_ at 10 min
Phillips et al. (2015)	Reduced rSO_2_	rSO_2_ normalised at 24 hours (60 and 120 min remained reduced)
Steinback et al. (2012)	Reduced rSO_2_	rSO_2_ normalised at 10 min
Uchida et al. (2020)	Reduced rSO_2_	rSO_2_ normalised at 13–16 min

Effects of hypoxia on regional cerebral tissue haemoglobin oxygen saturation (rSO_2_) are compared to a normoxic baseline (i.e. either immediately before the hypoxic intervention or during a separate trial), unless otherwise indicated.

#### EEG

Two studies measured EEG indices during recovery from hypoxia (Table 6). One study reported differences in EEG indices persisted for up to 4 hours with no difference between 21% and 100% oxygen recovery [5], whereas another study reported rapid normalisation (minutes) of EEG indices albeit a transient (seconds) worsening with 100% oxygen breathing during recovery compared with 21% oxygen [19].

**Table 6:** Studies reporting recovery of EEG responses following a hypoxic exposure.

Reference	Effect of hypoxia	Recovery from hypoxia
Blacker et al. (2021)	Reduced MMN mean amplitude; no effect for MMN peak latency; increased P3a mean amplitude; no effect for P3a peak latency	MMN mean amplitude normalised at 120 min (reduced at 0, 20 and 60 min); shorter MMN peak latencies normalised at 240 min (reduced at 20, 60, 120 and 180 min); P3a mean amplitude normalised immediately for 21% and 100% O_2_ with no differences between 21% and 100% O_2_
Malle et al. (2016)	Overall, no effect for EEG spectral power for all groups; however, SEF95 increased	*Transient increase in slow-wave activity for both groups, including increased delta wave activity (first 16 sec, both groups) and theta wave activity (first 32 sec in 100% O_2_); SEF95 transiently increased in 21% O_2_ and decreased in 100% O_2_, with SEF95 lower in 100% compared with 21% O_2_ during first 32 sec and from 64–80

Effects are compared to a normoxic baseline (i.e. either immediately before the hypoxic intervention or during a separate trial), unless otherwise indicated.

*Comparisons were between conditions (not to baseline). Abbreviations: MMN = mismatch negativity component; SEF95 = the 95^th^ percentile spectral edge frequency (i.e. the frequency below which 95% of total electroencephalogram power was contained); EEG = electroencephalogram; O_2_ = oxygen.

## Discussion

### State and limitations of evidence

The purpose of this systematic review was to consolidate the available evidence on the recovery of cognitive and basic physiological responses following an acute hypoxic exposure. This was to improve our understanding of how hypoxia could impair performance and compromise safety despite returning to normoxic (or hyperoxic) air breathing. Currently, there are insufficient published articles to accurately quantify post-hypoxia recovery profiles of cognitive and physiological indices, and their influential factors, which prevented meta-analysing the data and restricted this article to a systematic review. Some studies also employed methodologies that made it difficult to extract true recovery durations and, therefore, the time point for full recovery could not be determined. This was often due to articles not reporting time course profiles (i.e. repeated measures) and only reporting a single value that represented a range of time. The majority of research (19 studies) included men and women to allow findings to be applicable to both sexes; however, there was still a tendency to favour males. Study quality was moderate (average Rosendal score of 58 ± 15) and methodological differences made it difficult to compare between studies. Nevertheless, there is sufficient evidence supporting the presence of a ‘hypoxia hangover’ and to help to inform post-hypoxia restrictions.

### Recovery of cognitive functions

Hypoxia exponentially degrades cognitive functions with increasing hypoxaemia until loss-of-consciousness [4]. During hypoxia, there is preferential blood flow to posterior regions of the brain that are essential for regulating vital functions (e.g. breathing) [41] but are not highly involved in complex cognitive functions. Therefore, the anterior regions are more at risk of reduced oxygen delivery and, since they are involved in higher order and more complex cognitive processes, are easily degraded by hypoxia. This may be exacerbated by increased oxygen requirement of active brain regions and higher neuronal sensitivity to oxygen deprivation. In the present review, studies included a range of simple and complex cognitive tasks, and since some of these were not sensitive to the effects of hypoxia, they were unable to provide insight into the recovery period. Nonetheless, there appears to be persistent cognitive impairments for some standardised cognitive tasks (or domains) and more complex simulated flight performance tasks.

The most informative study designs used repeated measures of simple tasks that have a short temporal resolution, such as reaction speed and attention (refer to Table 2). Initially, Phillips et al. (2009) reported choice reaction time was slower 10 min into recovery, with some participants exhibiting a slower reaction time compared to hypoxia [21]. The same researchers later demonstrated simple and choice reaction speed were slower after 1 and 2 hours into recovery, and normalised at 24 hours [20]; however, there were no measures between 2 and 24 hours, with full recovery likely occurring earlier. More recently, Blacker & McHail (2021) reported vigilant attention (i.e. mean reaction time using a 10 min psychomotor vigilance task) was reduced 20 min into recovery and normalised after 1 hour, with no differences between breathing 21% or 100% oxygen during recovery [5]. This was similar to an earlier study that reported total response time for simple and choice reaction time tasks normalising by 60 min [17]. These observations suggest that performance of both simple reaction time and more demanding vigilance tasks can be impaired in the hours following a hypoxic exposure.

Performance of complex tasks were less informative as these are more difficult to measure. Since they typically take longer to assess, repeated measures are also difficult to ascertain. Similarly, studies reporting cognitive function for a single timepoint or range of time proximal to the cessation of hypoxia did not provide much insight into cognitive recovery profiles. Nevertheless, auditory serial addition task performance (a measure of attention and working memory) normalised at 90 sec [19], and task time and errors for serial number reading using the King-Devick test normalised at 5 min [25], suggesting rapid recovery. There may also be differences in sensory processing following hypoxia. For example, auditory monitoring tasks may recover at a slower rate compared to visual monitoring, memory and mathematical processing [13]. Future studies should aim to corroborate these findings and discern if there is a difference in post-hypoxia recovery profiles between simple and complex cognitive tasks, and how these are influenced by the hypoxic dose and recovery procedures.

Flight performance tasks are more reflective of the real-world implications posed by post-hypoxia cognitive impairments. Robinson et al. (2018) assessed simulated flight performance in non-pilots concurrently with a time-estimation task during successive hypoxic exposures, but only comparisons between different recovery modalities were possible. Following hypoxic exposure (i.e. normobaric 6.5% oxygen for 5 min), there were no differences in flight and time-estimation task performance between 21% and 13.1% oxygen breathing after 35 min [22]. However, flight performance errors and time-estimation task lapses during recovery with 13.1% oxygen were higher when preceded by hypoxia compared with normoxia, suggesting there was an additive effect from the prior hypoxic exposure [22]. Further, Varis et al. (2019 & 2022) assessed simulated flight performance during a return-to-base landing following an inflight hypoxic emergency on trained Hawk fighter pilots in two studies and reported impairments persisted for 10 min following exposure to 6% oxygen, alongside impaired situational awareness and adverse subjective feelings such as light headedness, visual impairments and dizziness [6, 28]. Some participants also reported incidents when driving home following testing [6].

### Recovery of physiological and neurophysiological status

Hypoxia elicits an integrated physiological response that stems primarily from peripheral chemoreceptors sensing hypoxaemia [42]. This chemoreflex is initiated from receptors predominantly located in the carotid body, which increase activity via the carotid sinus nerve that projects to the lower brainstem and nucleus tractus solitarius. This increases ventilation and autonomic sympathetic activity to raise HR and BP, and redistribute blood flow to critical tissues where vasodilation occurs, such as the brain [43]. The subsequent baroreflex initiates a vagal response and there seems to be a baroreflex resetting to shift baroreceptor activity to a higher threshold that allows for sustained and increased sympathetic innervation [36]. In severe hypoxia, compensatory responses are insufficient to maintain brain tissue oxygenation [44], which results in widespread slowing of EEG indices [19]. During normoxic recovery, HR and SpO_2_ recover within seconds and only marginally faster when breathing 100% oxygen (refer to Table 3). This withdraws the primary chemoreceptor stimulus, yet some physiological perturbations persist.

Autonomic nervous system activity can be inferred using cardiac indices, such as HRV. During hypoxia, HRV indices tend to decline, including SDNN, rMSSD and LF and HF spectral domains (refer to Table 3), indicating greater sympathetic innervation and parasympathetic withdrawal; however, these seem to normalise within 3–20 min [14, 15, 19, 23]. Sex and susceptibility to hypoxia also appear to influence the HRV response. For example, Botek et al. (2015) reported greater vagal withdrawal (i.e. lower HF spectral domain) during hypoxia and at 7 min into recovery in participants exhibiting a lower SpO_2_ [14]. The same researchers later reported males had a relatively higher sympathetic response to hypoxia exposure compared with females (i.e. higher natural logarithm of SDNN/rMSSD and LF spectral domain), but differences did not persist at 7 min into recovery [15]. Therefore, HRV indices suggest the increase in sympathetic activity during hypoxia normalises minutes into recovery.

Increased sympathetic activity may, in fact, persist for longer than indicated by HRV indices. For example, several included articles also reported increased MSNA for at least 15–20 min into recovery [26, 33, 35, 36, 38]. Although MNSA was not included in this review due to it being a difficult measure to ascertain, it provides a direct measure of sympathetic activity and highlights that increased sympathetic activity during hypoxic recovery may not be fully captured by HRV. The reasons for this are uncertain and despite elevated post-hypoxia MNSA being reduced by periods of hyperoxic breathing to suggest reduced chemoreflex activity [35], the rapid normalisation of SpO_2_, end-tidal gases and VE indicates chemosensitivity is unlikely underpinning persistent elevated MSNA. Rather, increased MSNA is more likely due to other reasons, such as long-term potentiation of post-ganglionic nerves [35].

The cardiovascular response to hypoxia increases BP, particularly SBP, which appears to normalise within 5–20 min in most, but not all, studies (refer to Table 3). Increased BP initiates the baroreflex to increase vagal activation and during hypoxia there is an attenuated cardiac baroreflex to allow for vagal adaptation. For example, Roche et al. (2002) demonstrated the increased hypoxic sympathetic excitation can be followed by an upregulated parasympathetic drive stemming from overactivity of the baroreflex to cause relative bradycardia during recovery [23]. This reduction in post-hypoxia HR was also shown by Botek et al. (2015 & 2018) [14, 15]. The interaction of arterial carbon dioxide and hypoxia may also alter baroreceptor resetting, with poikilocapnic hypoxia potentially having less influence on baroreceptor resetting compared to isocapnic hypoxia [45]. These effects highlight a complex interplay of chemoreflex and baroreflex regulation during hypoxia and recovery.

Brain tissue oxygenation (e.g. rSO_2_) is a more informative measure than peripheral indices (e.g. SpO_2_) of cognitive function [44]. However, studies measuring post-hypoxia rSO_2_ using near-infrared spectroscopy (fNIRS) report conflicting findings. Phillips et al. (2009 & 2019) demonstrated rSO_2_ remained reduced at 10 min [21] and 2 hours [20], and normalised by 24 hours into recovery [20]. These perturbations in rSO_2_ mirrored performance impairments for SRT and CRT tasks [20, 21]. Nonetheless, considering no measures were taken between 2 and 24 hours, rSO_2_ probably normalised before 24 hours and, as these studies were not adequately controlled, the authors described the findings as preliminary. In comparison, Steinback et al. (2012) and Uchida et al. (2020) reported rSO_2_ normalised 10–16 min into post-hypoxia recovery [27, 39]. These differences are difficult to explain but are likely due to methodological differences in hypoxic and recovery interventions, and fNIRS measurement techniques, which may not necessarily accurately reflect brain tissue oxygenation [46]. Further, measures of frontal/prefrontal cortex oxygenation represents a tissue average and may not be sensitive to regional differences that can occur during hypoxia.

The ventilatory increase during hypoxia peaks after 5 min then declines over 20–30 min to a steady-state above pre-hypoxic levels [26]. This hyperventilation can cause hypocapnia if carbon dioxide is not administered within the breathing gas (i.e. poikilocapnic hypoxia), which may be an important physiological determinant in studies measuring flight performance during hypoxic recovery [28]. Ventilation typically normalised within 15 min following cessation of hypoxia (refer to Table 4) and, in some studies, this included an initial transient undershoot to below pre-hypoxic levels after returning to normoxia [40] and hyperoxia (compared to hyperoxic baseline) [16]. The ventilatory response to repeated hypoxic exposures may also be attenuated [31], which suggests central chemosensitivity is reduced, which could increase susceptibility to hypoxia by exacerbating hypoxaemia.

EEG measures provide insight into brain signalling activity. Malle et al. (2016) measured EEG waveforms whilst performing a demanding cognitive task (i.e. PASAT) and showed an increase in SEF95 during hypoxia (i.e. the frequency below which 95% of total EEG power was contained), suggesting an increase in fast-wave activity [19]. However, recovery with 100% oxygen breathing generated a robust EEG slowing for ~30 sec (i.e. increase in theta activity and decrease in SEF95) [19], suggesting hyperoxia may elicit an initial harmful effect on the brain. Whereas, Blacker & McHail (2021) measured passive elicited event-related potentials that assess auditory processing, and demonstrated a continued decline in mismatch negativity (MMN) amplitude during post-hypoxia recovery, which normalised after 120 min, and a delayed response MMN peak latency, with shorter latencies that normalised after 240 min [5]. Whereas, P3a, a measure of attention, normalised immediately during recovery, and 100% compared with 21% oxygen breathing had no effect on recovery of EEG indices [5]. Therefore, pre-conscious auditory processing may require up to 2 hours to recover following a hypoxic exposure, which could be connected with the persistent impairment in auditory monitoring previously reported [13].

### Perspectives and conclusion

Understanding recovery from hypoxia and its practical implications is critical for various populations. Current research suggests there may be lagging effects for the recovery of some cognitive and physiological indices, but temporal profiles are unclear. Generally, most research has focussed on measuring responses to hypoxia rather than during recovery following return to normoxic and/or hyperoxic breathing. The effects of hypoxia have largely been established and although there is a better need to understand how these affect behaviour and decision making within various real-world situations, further research also needs to consider the post hypoxic period. Impaired cognitive functions can compromise performance and safety, and there is emerging evidence to suggest complex real-world skills, such as piloting an aircraft, are impaired during the immediate minutes following a hypoxic exposure. Thus, if there is the assumption that recovery is rapid due to SpO_2_ normalising within seconds-to-minutes, then individuals are likely to expose themselves to unnecessary risk during the post-hypoxia period that could result in serious or fatal incidents. This could occur following numerous situations, such as an inflight decompression at high-altitude, failure of oxygen supply systems to provide sufficient oxygen, hypoxia recognition training (i.e. standard military aircrew training), or after hypoxia research studies. However, the duration of potential impairments and the recovery profiles are unclear.

The mediating effects of the hypoxic dose and level of oxygen supplied during recovery is also uncertain. It seems plausible that the longer and more severe a hypoxic exposure, the longer the recovery period. However, whether interactions of F_I_O_2_ (and/or barometric pressure) and duration that elicit similar hypoxic doses cause different recovery profiles should also be assessed. For example, studies within this review included prolonged moderate-hypoxia (e.g. ~18,000 ft [5,486 m] equivalent or ~10–11% oxygen) and short severe-hypoxia (e.g. ~25,000 ft [7,620 m] equivalent or ~7–8% oxygen) interventions, but it is unclear which elicits greater cognitive and physiological effects during the post-hypoxic recovery. With poikilocapnic hypoxia, there is also an increased risk of hypocapnia resulting from hyperventilation, which causes cerebral hypoperfusion to exacerbate the effects of hypoxia. Therefore, the mediating effect of adding carbon dioxide to the recovery gas also needs to be examined.

Hyperoxic breathing is currently a focal area of research, particularly in military aviation, and appears to have a beneficial effect on aspects of cognition [47]. Nonetheless, the use of 100% oxygen for recovery from hypoxia did not demonstrate a beneficial effect compared to normoxia. Rather, 100% compared with 21% oxygen breathing during recovery seemed to cause an EEG slowing and impaired cognitive performance during the initial seconds following hypoxia [19], but this was not reported for all studies [5]. This suggests hyperoxic recovery may be harmful, which has previously been eluded to in the military aviation context [4], yet remains standard practice. There may also be a paradoxical effect whereby hyperoxic or nomoxic breathing post-hypoxia elicits a transient worsening of cognitive functions and symptoms, termed the ‘oxygen paradox’ [48]. This is potentially due to a transient vasoconstrictive effect of hyperoxia that reduces cerebral perfusion and a drop in arterial blood pressure [49], thus exacerbating tissue hypoxia. However, to the authors’ knowledge, there is no published literature demonstrating the effects of hyperoxia compared with normoxia on post-hypoxia cerebral perfusion and tissue oxygenation. In fact, there is scant research investigating the oxygen paradox, including cognitive, physiological and perceptive responses.

Whether hypoxia is induced by a reduction in barometric pressure or F_I_O_2_ is critical to manage the risk of decompression sickness (DCS). Ascending above 18,000 ft (5,485 m), which is approximately half the atmospheric pressure at sea-level, is associated with an increased risk of venous gas emboli and DCS [50]. Although we did not include DCS risk within our review, this is an important consideration in the aviation context and pose a greater risk than hypoxia alone.

In summary, this systematic review suggests there is a need for post-hypoxia restrictions to minimise the risk of potential incidents and accidents due to lagging cognitive and physiological effects. As such, current evidence supports the presence of a ‘hypoxia hangover’ but the severity and duration of impairments are difficult to quantify and likely depend on several factors. Future research should aim to systematically assess a range of cognitive and physiological responses that persist into recovery following a hypoxic exposure, and the influence of different conditions (e.g. hypoxic dose and recovery procedures). This review suggests that recovery of SpO_2_ and HR may only indicate partial recovery, and normalisation of other physiological indices can require to up to ~2–4 hours to return to levels before the hypoxic exposure. It therefore seems appropriate that safety measures are implemented following acute hypoxic exposures to mitigate the risks imposed by persistent cognitive impairments and physiological perturbations.

## Supporting information

S1 Table Methodological quality assessment summary. (DOCX)

## Abbreviations

BP: Blood pressure
CO_2_: Carbon dioxide
DBP: Diastolic blood pressure
DCS: Decompression sickness
ECG: Electrocardiogram
EEG: Electroencephalogram
F_I_CO_2_: Fraction of inspired carbon dioxide
HR: Heart rate
HRV: Heart rate variability
LF/HF: Low-frequency/high-frequency
MAP: Mean arterial pressure
MMN: Mismatch negativity component
MSNA: Muscle sympathetic nerve activity
NASA-TLX: National Aeronautics and Space Administration-Task Load Index Task load index
O_2_: Oxygen
PASAT: Paced Auditory Serial Addition Task
P_ET_CO_2_: End-tidal partial pressure of carbon dioxide
P_ET_O_2_: End-tidal partial pressure of oxygen
PO_2_: Partial pressure of oxygen
PRISMA: Preferred Reporting Items for Systematic Reviews and Meta-Analysis
PVT: Psychomotor vigilance task
rMSSD: Root mean square of successive differences between normal heartbeats
rSO_2_: Regional cerebral tissue haemoglobin oxygen saturation
SBP: Systolic blood pressure
SDNN: Standard deviation of NN intervals
SpO_2_: Peripheral blood haemoglobin-oxygen saturation
SYNWIN: Synthetic work environment
VE: Minute ventilation
