# Correction: Recovery from acute hypoxia: A systematic review of cognitive and physiological responses during the ‘hypoxia hangover’

**DOI:** 10.1371/journal.pone.0319046

**Published:** 2025-02-05

**Authors:** David M. Shaw, Peter M. Bloomfield, Anthony Benfell, Isadore Hughes, Nicholas Gant

There is an error in the description of the Hypoxic and Recovery Interventions for the articles Beer et al. (2017) and Dart et al. (2017) in Table 1. Please see the correct Table 1 here.

**Table 1:** Characteristics of included studies reporting recovery of cognitive and/or physiological responses following a hypoxic exposure.

Reference	Population	Hypoxic and Recovery Interventions
**Bascom et al. (1992)**	*n* = 6 (4 men, 2 women), mean age = 24.5, healthy adults	*Method*: Mouth-piece *Baseline*: 10 min normobaric O_2_ to P_ET_O_2_ of 100 mmHg, with P_ET_CO_2_ held at 1–2 mmHg above resting *Hypoxia*: 20 min reduced normobaric O_2_ to P_ET_O_2_ of 45, 50, 55, 65 or 75 mmHg, with P_ET_CO_2_ held at 1–2 mmHg above resting *Recovery*: 5 min normobaric O_2_ to P_ET_O_2_ of 100 mmHg, with P_ET_CO_2_ held at 1–2 mmHg above resting *Note*: The second hypoxia period was omitted.
Beer et al. (2017)^#^	*n* = 11 (9 men, 3 women; *n* = 1 dropout but sex not specified), healthy military personnel	*Method*: Hypobaric chamber and mask *Baseline*: 15 min normobaric 21% O_2_, then ascent to 10,000 ft at 5,000 ft/min, 9 min 10,000 ft, then 30 min 100% O_2_ at 10,000 ft *Hypoxia*: Ascent to 18,000 ft or 25,000 ft at 5000 ft/min, then maintained for 20 min or (typically) <5 min, respectively *Recovery*: 100% O_2_ during descent to ground level at 5000 ft/min for SpO_2_ >90%, then 15 min normobaric 21% O_2_
Blacker et al. (2021)	*n* = 26 (14 men, 12 women; *n* = 3 excluded due to noisy EEG data but sex not specified), mean age = 30.5, healthy Air Force personnel	*Method*: Reduced oxygen breathing environment and mask *Baseline*: 30 normobaric 21% O_2_ *Hypoxia*: 10 min normobaric 9.7% O_2_ (~20,000 ft equivalent) *Recovery*: 30 min normobaric 21% or 100% oxygen, then 210 min (mask-off) normobaric 21% O_2_
Botek et al. (2015)	*n* = 29, men, mean age = 26, healthy sports science students	*Method*: Mask *Baseline*: 7 min (mask off) normobaric 21% O_2_ *Hypoxia*: 10 min normobaric 9.6% O_2_ (~20,341 ft equivalent) *Recovery*: 7 min (mask-off) normobaric 21% O_2_
Botek et al. (2018)	*n* = 58 (28 men, 30 women), mean age = 23.8, healthy students	*Method*: Mask *Baseline*: 6 min (mask off) normobaric 21% O_2_ *Hypoxia*: 10 min normobaric 9.6% (~20,341 ft equivalent) *Recovery*: 7 min normobaric 21% O_2_
**Dahan et al. (1995)**	*n* = 10 (6 men, 4 women), age range = 23 to 32, healthy adults	*Method*: Mask *Baseline*: 15–20 min normobaric O_2_ to P_ET_O_2_ 109 mmHg (i.e. normoxic) or normobaric O_2_ to P_ET_O_2_ 525 mmHg (i.e. hyperoxic) *Hypoxia*: 30 sec or 1, 3, and 5 min reduced normobaric O_2_ to P_ET_O_2_ 49 mmHg *Recovery*: 3–5 min normobaric 21% O_2_ (3 and 5 min hypoxic exposures were administered an additional 10 min normobaric 70% O_2_) or 3–5 min hyperoxia (P_ET_O_2_ 525 mmHg)
Dart et al. (2017)^#^	*n* = 10, men, mean age = 31.4, healthy military personnel	*Method*: Hypobaric chamber and mask *Baseline*: 15 min normobaric 100% O_2_ prior to the 10,000 ft and 15,000 ft exposures and 30 min normobaric 100% O_2_ prior to the 20,000 ft exposure *Hypoxia*: ~60 min, ~45 min, and ~20 min at 10,000 ft, 15,000 ft, and 20,000 ft, respectively *Recover*y: 100% O_2_ during descent to ground level, then 75 min (mask-off) 21% normobaric O_2_
Easton et al. (1988)	*n* = 11 (5 men, 6 women), mean age = 25, healthy adults	*Method*: Mouth-piece *Baseline*: 6–8 min normobaric 21% O_2_ *Hypoxia*: 25 min normobaric 8–10% O_2_ to SpO_2_ of ~80% with P_ET_CO_2_ held constant to baseline *Recovery*: Either; 1) 7 min normobaric 21% O_2_; 2) 15 min normobaric 21% O_2_; 3) 60 min normobaric 21% O_2_; 4) 7 min normobaric 100% O_2_; or 5) 15 min normobaric 30% O_2_, *Note*: The second hypoxic period was omitted
Georgopoulos et al. (1990)	*n* = 8 (5 men, 3 women), mean age = 28, healthy adults	*Method*: Mouth-piece *Baseline*: 8–10 min normobaric 21% O_2_, then 5 min 21% O_2_ with added CO_2_ (i.e. F_I_CO_2_ of 4.5–5%; P_ET_CO_2_ 5–14 mmHg), then 5 min 21% O_2_ *Hypoxia*: 25 min normobaric 8–9% O_2_ to SpO_2_ of ~80% (or 21% O_2_) with P_ET_CO_2_ held constant to baseline *Recovery*: 5 min normobaric 21% O_2_, then 5 min 21% O_2_ with added CO_2_ (i.e. CO_2_ titrated to match P_ET_CO_2_ of initial hypercapnic exposure), then 5 min 21% O_2_
Harshman et al. (2015)	*n* = 8, men, mean age = 29.9, healthy military personnel	*Method*: Mask *Baselin*e: 5 min normobaric 21% O_2_ *Hypoxia*: 5 min normobaric 8% O_2_ *Recovery*: 5 min normobaric 100% O_2_
Janaky et al. (2007)	*n* = 14, men, mean age = 39.7, health adults, either candidates for pilot school or lab personnel	*Method*: Hypobaric chamber *Baseline*: 20 min normobaric 21% O_2_ *Hypoxia*: 15 min at 5500 m (18,044 ft) *Recovery*: 20 min normobaric 21% O_2_
Malle et al. (2016)	*n* = 42, men, mean age = 29.4, healthy adults	*Method*: Mask *Baseline*: 30 sec normobaric 21% O_2_ or 100% O_2_ (normoxia included 10 min prior 21% O_2_ and hyperoxia included 3 min prior 100% O_2_) *Hypoxia*: ~3 min normobaric 6% O_2_ (or 21% O_2_) *Recovery*: 10 min normobaric 21% O_2_ or 3 min 100% O_2_ then 21% O_2_ *Note*: n = 22 in Hypoxia-Air group and *n* = 20 in Hypoxia-O_2_ group; comparisons between group and to baseline
Morgan et al. (1995)	*n* = 10, men, mean age = ~37, healthy adults	*Method*: Mask *Baseline*: Normobaric 21% O_2_ *Hypoxia*: 20 min reduced normobaric O_2_ to SpO_2_ 80% and increased CO_2_ to P_ET_CO_2_ to +5 mmHg of baseline (control group of *n* = 5 breathed 21% O_2_ for 40 min) *Recovery*: 20 min normobaric 21% O_2_
Najmanová et al. (2019)	*n* = 38 (15 men, 23 women), mean age = ~25, healthy adults	*Method*: Mask *Baseline*: 7 min normobaric 21% O_2_ *Hypoxia*: 10 min normobaric 9.6% O_2_ (~20,341 ft equivalent) *Recovery*: 7 min normobaric 21% O_2_
**Phillips et al. (2009)**	*n* = 36, healthy military aviation personnel	*Method*: Mask *Baseline*: 10 min 21% O_2_ *Hypoxia* (profile 1, *n* = 9): 3 rapid (2–3 s) transitions to normobaric 9.1, 11.4 and 14.1% O_2_ with rapid return to 21% O_2_ between each (i.e. 5 min at altitude and 5 min 21% O_2_), then gradual transition (4 min) to normobaric 9.1% O_2_ and return to 21% O_2_ *Hypoxia* (profile 2, *n* = 7): Gradual transition (4 min) to normobaric 9.1% O_2_ and return to 21% O_2_, then 3 rapid (2–3 s) transitions to normobaric 9.1, 14.1, and 11.4% O_2_ with return to 21% O_2_ between each (i.e. 5 min at altitude and 5 min 21% O_2_) *Note*: *n* = 20 were in the control (normoxic) group. *Recovery*: Gradual (4 min) transition to normobaric 21% O_2,_ then maintained for 10 min
**Phillips et al. (2015)**	*n* = 19, healthy active duty military personnel	*Method*: Mask *Baseline*: 21% normobaric O_2_ on prior visit *Hypoxia*: 30 min (or until SpO_2_ dropped below 50%) normobaric 9.96% O_2_ (~18,000 ft equivalent) *Recovery*: 24 hr normobaric 21% O_2_
Querido et al. (2010)	*n* = 7 (5 men, 2 women), mean age = 30, healthy	*Method*: Mask *Baseline*: ≥15 min normobaric 21% O_2_ *Hypoxia*: 20 min reduced O_2_ to SpO_2_ 80% and increased CO_2_ to P_ET_CO_2_ to baseline levels *Recovery*: ~4.5 min normobaric 21%, then 1 min normobaric 100% O_2_ (termed “intervention” and repeated 3 times)
**Querido et al. (2011)**	*n* = 14 (11 men, 3 women), mean age = 26, healthy adults	*Method*: Mask *Baseline*: 15 min normobaric 21% O_2_ *Hypoxia*: 20 min of reduced normobaric O_2_ to SpO_2_ of 80% with P_ET_CO_2_ maintained at baseline levels *Recovery*: ~5 min normobaric 21% O_2_ (n = 15) and 20 min normobaric 21% O_2_ (*n* = 5)
**Robinson et al. (2018)**	*n* = 21 (20 men, 1 women), age range = 22–37, healthy Air Force personnel (non-pilots)	*Method*: Mask *Baselin*e: 5 min normobaric 21% O_2_ *Hypoxia*: 5 min normobaric 6.5% O_2_ (~25,000 ft equivalent) *Recovery*: 5 min normobaric 21% O_2_ followed by 30 min normobaric 21% or 13.1% O_2_ (~10,000 ft equivalent) *Note*: Comparisons with baseline were not possible due to methodological issues
**Roche et al. (2002)**	*n =* 11 (6 men, 5 women), mean age = 29, healthy adults	*Method*: Mask *Baseline*: 20 min normobaric 21% O_2_ *Hypoxia*: 15 min normobaric 11% O_2_ (~15,748 ft equivalent) *Recovery*: 20 min normobaric 21% O_2_
**Sausen et al. (2003)**	*n* = 4 (3 men, 1 women), mean age = 24.7, healthy US student Naval flight surgeons	*Method*: Mask *Baseline*: 15 min normobaric 21% O_2_ *Hypoxia*: 20 min transition to normobaric 10% O_2_ (~18,000 ft equivalent), then 30 min 10% O_2_, then 5 min transition to normobaric 21% O_2_ *Recovery*: 15 min normobaric 21% O_2_
Steinback et al. (2012)	*n* = 7 (3 men, 4 women), mean age = 27, healthy adults	*Method*: Mask *Baseline*: 10 min normobaric 21% O_2_ *Hypoxia*: Step decrease in normobaric O_2_ to P_ET_O_2_ of 45 mmHg (~80% SpO_2_) and maintenance of baseline P_ET_CO_2_, then maintained for 5 min *Recovery*: 10 min normobaric 21% O_2_
Stepanek et al. (2013)	*n* = 25 (14 men, 11 women), mean age = 32.4, healthy adults	*Method*: Mask *Baseline*: Normobaric 21% O_2_ *Hypoxia*: ~4–5 min normobaric 8% O_2_ (~23,300 ft equivalent) *Recovery*: ~4–5 min normobaric 21% O_2_
Stepanek et al. (2014)	*n* = 25 (14 men, 11 women), mean age = 32.4, healthy adults	*Method*: Mask 2 profiles separated by a 6 min washout period *Baseline*: Normobaric 21% O_2_ *Hypoxia*: 3 min either normobaric 8% O_2_ or normobaric 7% O_2_ + 5% CO_2_ *Recovery*: 3 min normobaric 21% O_2_
Tamisier et al. (2005)	*n* = 10 (6 men, 4 women), mean age = 29.8, healthy adults	*Method*: Mask Baseline: Normobaric 21% O_2_ Hypoxia: 2 hr normobaric ~9% O_2_ to ~85% SpO_2_ Recovery: Normobaric 21% O_2_
Uchida et al. (2020)	*n* = 9 (2 men, 7 women), mean age = 28, healthy adults	*Method*: Mask *Baseline*: 7 min normobaric 21% O_2_ *Hypoxia*: 7–10 min normobaric 11.8% O_2_ (~15,00 ft equivalent) *Recovery*: 13–16 min normobaric 21% O_2_
**Varis et al. (2019)**	*n* = 16, men, healthy qualified Hawk pilots	*Method*: Mask 3 profiles separated by 10 min washout periods within ~40 min *Baseline*: N/A *Hypoxia*: Pressurised air, then ~123 sec, ~93 sec, and ~115 sec of normobaric 8% (~20,341 ft equivalent), 7% (~22,966 ft equivalent) or 6% O_2_ (~25,919 ft equivalent), respectively *Recovery*: 100% normobaric O_2_ until emergency procedure completion; return to base flight 10 min after emergency procedures completion *Note*: Performance was compared to control flight with normobaric 21% O_2_ (not hypoxia) following the 6% O_2_ condition only; 60 min rest between trials
**Varis et al. (2022)**	*n* = 15, men, mean age = 24.6, healthy Hawk fighter pilots	*Method*: Mask *Baseline*: N/A *Hypoxia*: ~75 sec normobaric 6% O_2_ or ~103 sec normobaric 8% O_2_ *Recovery*: ~58 sec normobaric 100% O_2_ following 6% O_2_ and ~42 sec normobaric 100% O_2_ following 8% O_2_ (to complete emergency procedures); return to base flight 10 min after emergency procedures completion *Note*: Performance was compared to control flight with normobaric 21% O_2_ (not hypoxia)
**Vigo et al. (2010)**	*n* = 12, men, mean age = 28, healthy military pilots	*Method*: Hypobaric chamber and mask *Baseline*: ~89 sec 100% O_2_ during ascent to 27,000 ft *Hypoxia*: ~113 sec at 27,000 ft *Recovery*: ~137 sec 100% O_2_ at 27,000 ft
**Xie et al. (2001)**	*SpO2*, *HR*, *BP and HRV*: *n* = 11 (7 men, 4 women), mean age = 26, healthy adults *Ventilation*: *n* = 9 (6 men, 3 women), mean age = 29, healthy adults	*Method*: Mask *Baseline*: 10 min normobaric 21% O_2_ *Hypoxia*: 20 min normobaric 10–12% O_2_ to lower SpO_2_ to ~80% and maintain baseline P_ET_CO_2_ *Recovery*: 20 min normobaric 21% O_2_

The estimated altitude equivalent is reported for studies using normobaric hypoxia that reported targeting a specific altitude. Altitude equivalents are reported in ft in line with aviation standards; for reference: 5,000 ft = 1,524 m, 10,000 ft = 3,048 m, 18,000 ft = 5,486 m, and 25,000 ft = 7,620 m. Abbreviations: O_2_ = oxygen; SpO_2_ = peripheral blood haemoglobin-oxygen saturation; P_ET_O_2_ = end-tidal partial pressure of oxygen; CO_2_ = carbon dioxide; F_I_CO_2_ = fraction of inspired carbon dioxide; P_ET_CO_2_ = end-tidal partial pressure of carbon dioxide. ^#^Amended description of the hypoxia and recovery interventions.

## References

[1] Shaw DM, Bloomfield PM, Benfell A, Hughes I, Gant N (2023) Recovery from acute hypoxia: A systematic review of cognitive and physiological responses during the ‘hypoxia hangover’. PLOS ONE 18(8): e0289716. 10.1371/journal.pone.0289716 37585402 PMC10431643

