# Supplementary material for: Recovery from acute hypoxia: A systematic review of cognitive and physiological responses during the ‘hypoxia hangover’
Source: PLoS One. 2023 Aug 16;18(8):e0289716. doi: 10.1371/journal.pone.0289716 (PMC10431643; doi:10.1371/journal.pone.0289716)
Supplement: S1 Table — (DOCX) [file pone.0289716.s001.docx]

S1 Table. Methodological quality assessment summary.

| **Reference** | A clear description of the inclusion and exclusion criteria was provided* | The trials were randomised | The method used to generate the random allocation sequence was described | Treatment order was counterbalanced | Sample size was justified | Attempts were made to control and/or monitor pre-trial conditions | Design incorporated measures of important baseline variables | Subjects were blinded | Methods and successfulness of blinding were described | Details were provided regarding the inability of a subject to complete study requirements | Statistical methods used to compare groups for primary outcome measure(s), and methods for additional analyses were described | Both point measures and measures of variability for the primary outcome were provided | The results of between groups statistical comparisons were reported for the primary outcome measures | Reproducibility of the primary outcome measure(s) was reported | A familiarisation of the performance test was conducted | **Total Score (%)** |
| --- | --- | --- | --- | --- | --- | --- | --- | --- | --- | --- | --- | --- | --- | --- | --- | --- |
| Bascom et al. (1992) | 0 | 1 | 0 | 0 | 0 | 0 | 1 | 0 | - | 1 | 1 | 1 | 1 | 0 | - | 42.9 |
| Beer et al. (2017) | 0 | 0 | - | 1 | 0 | 1 | 1 | 0 | - | 1 | 1 | 1 | 1 | 0 | 1 | 61.5 |
| Blacker et  al. (2021) | 1 | 0 | - | 1 | 0 | 0 | 1 | 0 | - | - | 1 | 1 | 1 | 0 | 0 | 50.0 |
| Botek et al. (2015) | 1 | - | - | - | 0 | 1 | 1 | - | - | - | 1 | 1 | 1 | 0 | - | 75.0 |
| Botek et al. (2018) | 1 | - | - | - | 0 | 1 | 1 | - | - | - | 1 | 1 | 1 | 0 | - | 75.0 |
| Dahan et al. (1995) | 0 | 1 | 0 | 0 | 0 | 1 | 1 | 0 | - | - | 1 | 1 | 1 | 0 | - | 50.0 |
| Dart et al. (2017) | 1 | 1 | 0 | 0 | 1 | 0 | 1 | 1 | - | 1 | 1 | 1 | 1 | 0 | 1 | 71.4 |
| Easton et al. (1988) | 0 | 1 | 0 | 0 | 0 | 0 | 1 | 0 | - | - | 1 | 1 | 1 | 0 | - | 41.7 |
| Georgopoulos et al. (1990) | 0 | 1 | 0 | 0 | 0 | 1 | 1 | 0 | - | - | 1 | 1 | 1 | 0 | - | 50.0 |
| Harshman et al. (2015) | 0 | 0 | - | 0 | 0 | 0 | 1 | - | - | - | 1 | 1 | 1 | 0 | - | 40.0 |
| Malle et al. (2016) | 0 | 1 | 0 | - | 0 | 0 | 1 | 0 | - | - | 1 | 1 | 1 | 0 | - | 45.5 |
| Morgan et al. (1995) | 0 | 0 | - | 0 | 0 | 0 | 1 | 0 | - | - | 1 | 1 | 1 | 0 | - | 36.4 |
| Najmanová (2019) | 1 | - | - | - | 0 | 1 | 1 | - | - | 1 | 1 | 1 | 1 | 0 | - | 77.8 |
| Phillips et al. (2009) | 0 | - | - | - | 0 | 0 | 1 | 0 | - | - | 0 | 1 | 0 | 0 | 1 | 30.0 |
| Phillips et al. (2015) | 1 | - | - | - | 0 | 1 | 1 | 0 | - | 0 | 1 | 1 | 1 | 0 | 1 | 63.6 |
| Querido et al. (2010) | 1 | - | - | - | 0 | 0 | 1 | - | - | - | 1 | 1 | 1 | 0 | - | 62.5 |
| Querido et al. (2011) | 1 | - | - | - | 0 | 1 | 1 | - | - | - | 1 | 1 | 1 | 0 | - | 75.0 |
| Robinson et al. (2018) | 1 | 1 | 1 | 1 | 1 | 1 | 1 | 1 | 0 | 1 | 1 | 1 | 1 | 0 | 1 | 86.7 |
| Roche et al. (2002) | 0 | - | - | - | 0 | 0 | 1 | - | - | - | 1 | 1 | 1 | 0 | 0 | 44.4 |
| Sausen et al. (2003) | 0 | - | - | - | 0 | 1 | 1 | 0 | - | - | 1 | 1 | 1 | 0 | - | 55.6 |
| Steinback et al. (2012) | 0 | - | - | - | 0 | 1 | 1 | - | - | - | 1 | 1 | 1 | 0 | - | 62.5 |
| Stepanek et al. (2013) | 1 | - | - | - | 1 | 0 | 1 | - | - | - | 1 | 1 | 1 | 0 | 1 | 77.8 |
| Stepanek et al. (2014) | 1 | 0 | - | 0 | 0 | 0 | 1 | 1 | 0 | - | 1 | 1 | 1 | 0 | - | 50.0 |
| Tamisier et al. (2005) | 0 | 1 | 0 | 0 | 0 | 1 | 1 | 0 | - | - | 1 | 1 | 1 | 0 | - | 50.0 |
| Uchida et al. (2020) | 1 | - | - | - | 0 | 1 | 1 | - | - | - | 1 | 1 | 1 | 0 | 1 | 77.8 |
| Varis et al. (2019) | 0 | - | - | 1 | 0 | 0 | 1 | 1 | 1 | - | 1 | 1 | 1 | 0 | 0 | 58.3 |
| Varis et al. (2022) | 0 | 1 | 0 | 0 | 0 | 0 | 1 | 1 | 1 | - | 1 | 1 | 1 | 0 | 0 | 50.0 |
| Vigo et al. (2010) | 0 | - | - | - | 0 | 0 | 1 | - | - | 1 | 1 | 1 | 1 | 0 | - | 55.6 |
| Xie et al. (2001) | 1 | - | - | - | 0 | 1 | 1 | - | - | - | 1 | 1 | 1 | 0 | - | 75.0 |

*A detailed description of participant characteristics was considered adequate.
